# Inferior Frontal Gyrus Volume Loss Distinguishes Between Autism and (Comorbid) Attention-Deficit/Hyperactivity Disorder—A FreeSurfer Analysis in Children

**DOI:** 10.3389/fpsyt.2018.00521

**Published:** 2018-10-23

**Authors:** Kathrin Nickel, Ludger Tebartz van Elst, Jacek Manko, Josef Unterrainer, Reinhold Rauh, Christoph Klein, Dominique Endres, Christoph P. Kaller, Irina Mader, Andreas Riedel, Monica Biscaldi, Simon Maier

**Affiliations:** ^1^Department of Psychiatry and Psychotherapy, Faculty of Medicine, Medical Center – University of Freiburg, Freiburg, Germany; ^2^Department of Child and Adolescent Psychiatry, Psychotherapy and Psychosomatics, Faculty of Medicine, Medical Center – University of Freiburg, Freiburg, Germany; ^3^Medical Psychology and Medical Sociology, Faculty of Medicine, University of Freiburg, Freiburg, Germany; ^4^Department of Neurology, Faculty of Medicine, Medical Center – University of Freiburg, Freiburg, Germany; ^5^Department of Neuroradiology, Faculty of Medicine, Medical Center – University of Freiburg, Freiburg, Germany

**Keywords:** autism spectrum disorder (ASD), attention-deficit/hyperactivity disorder (ADHD), FreeSurfer, cortical thickness, mean curvature

## Abstract

**Objective:** Autism spectrum (ASD) and attention-deficit/hyperactivity disorder (ADHD) are neurodevelopmental disorders with a high rate of comorbidity. To date, diagnosis is based on clinical presentation and distinct reliable biomarkers have been identified neither for ASD nor ADHD. Most previous neuroimaging studies investigated ASD and ADHD separately.

**Method:** To address the question of structural brain differences between ASD and ADHD, we performed FreeSurfer analysis in a sample of children with ADHD (*n* = 30), with high-functioning ASD (*n* = 14), with comorbid high-functioning ASD and ADHD (*n* = 15), and of typically developed controls (TD; *n* = 36). With FreeSurfer, an automated brain imaging processing and analyzing suite, we reconstructed the cerebral cortex and calculated gray matter volumes as well as cortical surface parameters in terms of cortical thickness and mean curvature.

**Results:** A significant main effect of the factor ADHD was detected for the left inferior frontal gyrus (Pars orbitalis) volume, with the ADHD group exhibiting smaller Pars orbitalis volumes. Dimensional measures of autism (SRS total raw score) and ADHD (DISYPS-II FBB-ADHD score) had no significant influence on the left Pars orbitalis volume. Both, ASD and ADHD tended to have an effect on cortical thickness or mean curvature, which did not survive correction for multiple comparisons.

**Conclusion:** Our results underline that ADHD rather than ASD is associated with volume loss in the left inferior frontal gyrus (Pars orbitalis). This area might play a relevant role in modulating symptoms of inattention and/or impulsivity in ADHD. The effect of comorbid ADHD in ASD samples and vice versa, on cortical thickness and mean curvature, requires further investigation in larger samples.

## Introduction

Previous studies mainly investigated autism spectrum disorder (ASD) and attention-deficit/hyperactivity disorder (ADHD) in isolation. The question to what extent there is a clinical overlap between ADHD and ASD, whether they represent distinct diagnostic categories or form a continuous disorder with a continuum of brain dysfunction remains open to discussion (1, 2). To identify endophenotypes across diagnostic categories, it is important to further investigate the overlap between ADHD and ASD on a behavioral, neurocognitive, and neurobiological level (3, 4).

### Co-occurrence of ASD and ADHD

ASD is characterized by impairment in social communication and interaction as well as by a range of stereotypic behaviors whereas deficits in attention, hyperactivity, impulsiveness, disorganization and affective instability represent the core symptoms of ADHD (5). As ASD is a frequent comorbid condition in ADHD and vice versa (1), there is the possibility of dual diagnosis according to DSM-5 (5). The interpretation of studies prior to 2013 is limited by DSM-IV guidelines not permitting the dual diagnosis of ASD and ADHD (6).

Studies report frequent co-occurrence with 30-50% of individuals with ASD manifesting ADHD symptoms (particularly at pre-school age) and two-thirds of individuals with ADHD showing features of ASD (7). Social difficulties in ADHD are often interpreted as part of ADHD symptoms rather than reflecting impairments in social communication being characteristic for ASD (8). Both disorders are highly heritable (9).

### Previous volumetric studies in ASD and ADHD

Previous structural neuroimaging studies encompassing children and adolescents with both, ADHD and ASD, are scarce and show heterogeneous results (Table 1). There is only one previous FreeSurfer study on ASD children with and without comorbid ADHD, but without a separate ADHD group (12). Mahajan et al. (12) found that gray matter (GM) volume and surface area (SA) were increased in the left postcentral and the right precentral gyrus which in this study was specific for ASD children without ADHD, whereas an increase in the left precentral gyrus was specific for children with ASD and comorbid ADHD. Regardless of ADHD comorbidity, all children with ASD showed increases in GM volume and SA in the left inferior parietal cortex (12).

**Table 1:** Previous volumetric studies comparing children with ADHD and ASD.

**Study**	**n (ADHD/ASD/TD)**	**Age in years (ADHD/ASD/TD)**	**IQ**	**Methods**	**Region(s) and results**
		**(Mean ± *SD*)**			
1. Brieber et al. (3)	15 ADHD 15 ASD 15 TD	13.13 ± 1.4 14.2 ± 1.9 13.3 ± 1.8 (10–16 y)	104.1 ± 15.8 106.8 ± 21.4 107.7 ± 12.7	VBM	Smaller GM in left medial temporal lobe and higher GM volume in left inferior parietal cortex in ADHD and ASD vs. TD Increased GM volume in right supramarginal gyrus in ASD vs. ADHD and TD
2. O'Dwyer et al. (10)	180 ADHD 140 TD 124 unaffected siblings	16.2 ± 3.7 16.8 ± 3.6 16.9 ± 4.0 (7.4–28.5 y)	98.8 ± 14.9 106.6 ± 13.2 101.8 ± 14.2	VBM	Increasing ASD score is associated with greater GM volume
3. Lim et al. (11)	44 ADHD 19 ASD 33 TD	13.6 ± 1.87 14.9 ± 1.86 14.3 ± 2.52	92.2 ± 11.7 113 ± 15.7 110 ± 11.5	VBM	Smaller right posterior cerebellar GM volume in ADHD vs. ASD and TD Larger left middle/superior temporal gyrus GM volume in ASD vs. ADHD and TD
4. Mahajan et al. (12)	30 ASD- 33 ASD+ 63 TD	10.5 ± 1.7 10.3 ± 1.4 10.5 ± 1.3 (8–12 y)	102 ± 14 103 ± 17 112 ± 11	Free Surfer ROI	Increased GM volume and SA in the left inferior parietal cortex in ASD+ and ASD- Increased GM volume and SA in the left post-central gyrus and the right precentral gyrus in ASD- Increased GM volume and SA in the left precentral gyrus in ASD+

*n, number; SD, standard deviation; ADHD, attention-deficit/hyperactivity disorder; ASD, autism spectrum disorder; ASD+, autism spectrum disorder with comorbid ADHD; ASD-, autism spectrum disorder without comorbid ADHD; TD, typically developed; VBM, voxel based morphometry; ROI, region of interest; GM, gray matter; IQ, intelligence quotient*.

Voxel based morphometry (VBM) studies display a heterogeneous picture. The most recent VBM study suggested GM reduction in the right posterior cerebellum to be disorder-specific for ADHD relative to ASD. GM enlargement in the middle/superior temporal gyrus, on the other hand, was reported to be disorder-specific for ASD relative to ADHD (11). An earlier VBM study pointed toward shared GM volume reduction within the medial temporal and higher GM in the inferior parietal cortex (3). Further, increased GM volume of the supramarginal gyrus was reported in ASD, but not ADHD, relative to controls (3). In the largest VBM study so far an increasing ASD score was associated with greater global GM volume (10).

In ASD it has frequently been reported that after having a normal (13) or smaller (14) brain size at birth, there is a period of early brain overgrowth prior to 4 years of age (14–16). The pathophysiology of such alterations is unknown, but it is proposed to result from deviant neuronal proliferation and axonal growth during fetal development that in turn leads to an aberrant developmental pruning (17). In contrast, in children with ADHD, smaller whole brain volumes (18–20) and lower GM volumes have been described (21). It is hypothesized that ADHD children show a delayed brain maturation process (22).

Despite similarities in clinical presentation as well as mutual comorbidity rates in ASD and ADHD, these disorders present a rather different neuroanatomical profile. Most studies report subcortical temporal structures such as the amygdala to be enlarged in young children (at ages 2–4) with ASD (23) with a normalization in late childhood and adolescence (24). Amygdala volumes in adults with ADHD have been found to be relatively normal (25, 26) or smaller than in controls (27). Basal ganglia are reported to be enlarged (15) in ASD and smaller in ADHD (1). With regard to the corpus callosum, thalamus and cerebellum, however, in many studies ASD and ADHD show a volume reduction (1).

### Previous cortical surface parameter studies in ASD and ADHD

Studies focusing on cortical surface parameters in ASD also reported mixed results. When investigating cortical surface parameters, it has to be reflected that cortical development in ASD varies across developmental stages or brain regions. Three different phases have been proposed: accelerated expansion in early childhood, accelerated thinning in later childhood and adolescence, and decelerated thinning in early adulthood (28). Hazlett et al. (29) examined young children with ASD (ages 2–5 years) and found increased cortical volumes, but no alterations in cortical thickness implicating that brain enlargement may be associated with increased cortical SA in ASD. Increased cortical thickness in temporal lobes was reported in children (ages 8–12 years) with ASD (30) with greater cortical thinning in ASD over time especially in occipital regions (31). Greater cortical thinning was associated with more severe symptoms in ASD (31). Further investigations pointed toward cortical thinning in adolescents (ages 12–25 years) with ASD (32–34). Studies in adults are divergent with some reporting cortical thinning (35–37) in brain regions involved in social cognition, others cortical thickening within frontal lobe regions (38) or regions from all four lobes (36). A large recent study found no significant difference in overall cortical thickness or surface area between ASD and typically developed (TD) (39).

In children and adults with ADHD, cortical thinning has been described in parietal and frontal regions responsible for executive function and attention (40–42). Another study detected no differences in cortical thickness of ADHD children, but decreased SA and cortical folding (43).

The unclear and puzzling current state requires further studies directly comparing volumetric and cortical thickness parameters between ASD and ADHD. Individuals with isolated autism and individuals, who present comorbid conditions in terms of ADHD, can be distinguished behaviorally as already documented by our research group (44).

### Rationale of our study

Based on the available evidence, we aimed to study the brain structure in children with ASD with and without comorbid ADHD as well as TD. To address the question of a potential neurobiological overlap between ADHD and ASD, we analyzed the brain scans for shared and disorder-specific abnormalities. We investigated differences in terms of GM as well as cortical thickness and mean curvature.

In doing so, this study represents the first FreeSurfer study comprising ASD and ADHD groups, as well as subjects with co-occurrence of both conditions. Because previous studies showed inconsistent and widely distributed changes, we did not limit the analysis to individual a priori regions of interest (ROIs).

## Materials and methods

### Participants

The ethics committee of the University Medical Center Freiburg approved the study (approval ID: 279/06). Magnetic resonance imaging (MRI) scans were acquired following written informed consent of the children's parents. Male children with ASD and ADHD were recruited from the Department of Child and Adolescent Psychiatry, Psychotherapy, and Psychosomatics of the University Medical Center Freiburg.

We obtained scans of high quality in 40 male children with a diagnosis of ASD according to ICD-10 and DSM-5 criteria. Twenty-nine ASD patients were included in the final analysis after 7 patients were excluded due to image artifacts, 3 due to IQ < 70 and one patient due to comorbid seizures. All ASD children were high-functioning and with no language delay. Intellectual disability (full scale IQ below 70), comorbid Tourette syndrome or severe neurological diseases were defined as exclusion criteria. With the exception of 2 patients, no ASD participant had comorbid depressive or anxiety symptoms. The diagnostic process followed international guidelines, including the Autism Diagnostic Observation Schedule [ADOS-G, (45)] and the Autism Diagnostic Interview [ADI-R, (46)]. Psychometric tools included the Child Behavior Checklist [CBCL, (47)], the Social Responsiveness Scale [SRS, (48)], and the diagnostic interview K-SADS-PL (49). According to the K-SADS-PL, 15 autistic children additionally met the diagnostic criteria for ADHD. The ADHD diagnosis was confirmed with the DISYPS-II FBB-ADHD (50), and verified by a multi-professional team of expert clinicians to ensure a comorbid ADHD. The group without (14 patients) and the group with comorbid ADHD (15 patients) were not significantly different for age and IQ.

Additionally, MRI-scans of 50 male patients with ICD-10 and DSM-5 ADHD diagnosis without a comorbid ASD were acquired. Twelve scans were excluded due to poor image quality, 7 due to the low IQ of the subjects (< 70) and one due to an arachnoid cyst, so that finally 30 ADHD patients were analyzed. ADHD diagnosis was clinically based on ICD-10 and DSM-5 criteria and additionally confirmed with the DISYPS-II FBB-ADHD (50). ASD symptoms were ruled out applying the SRS score (48), also the CBCL (47) was consulted. With the exception of one ADHD patient, no one suffered from comorbid depression or anxiety disorder as assessed by the K-SADS-PL (49). Methylphenidate medication in children with an ADHD diagnosis was discontinued at least 24 h prior to scanning procedure.

Fourty-eight typically developed male children (TD) were recruited from local schools and sport groups. Control subjects were included after a phone interview with the parents who additionally completed a sociodemographic questionnaire, the CBCL (47) and the SRS (48) for ruling out ASD and ADHD symptoms. Four children were excluded from the TD group, because of the presence of ADHD or autistic symptoms, 7 due to imaging artifacts and one due to an IQ < 70, so that we finally included 36 male TD participants in the study.

Subjects were matched according to IQ assessed with Raven's Standard Progressive Matrices (51), age and sex.

All subjects included in the study accomplished behavioral tasks of executive functions and planning as well. The results are published elsewhere (44).

### Image acquisition

A standard magnetization-prepared rapid gradient echo (MPRAGE) T1-weighted anatomical scan was conducted (relaxation time = 2,200 ms, echo time = 2.15 ms, flip angle = 12°, inversion time = 1,100 ms) on a 3T Siemens TIM Trio Magnetom scanner (Erlangen, Germany). Slice thickness was 1 mm and voxel size 1 × 1 × 1 mm^3^.

### Brain segmentation

Cortical reconstruction and segmentation was performed using FreeSurfer version 5.3 (http://surfer.nmr.mgh.harvard.edu/). FreeSurfer is a fully automated suite of tools that enables analysis of key features in the human brain such as segmentation of most macroscopically visible brain structures (52). FreeSurfer allows to compute the volume of subcortical areas and reconstructs the cerebral cortex (53). It also provides information about mapping of cortical GM thickness (54) and the construction of surface models of the cerebral cortex (55). The technical details of FreeSurfer procedures are described elsewhere (52). Applying FreeSurfer, we removed non-brain tissue and segmented cortical and subcortical GM and WM depending on image intensity. FreeSurfer output was inspected by three blinded trainees and rated on a scale ranging from 1 to 4. A “1” means no visible artifacts, whereas “4” denotes distinct blurred and low-quality images. Manual correction followed recommendations of FreeSurfer developers (https://surfer.nmr.mgh.harvard.edu/fswiki/FsTutorial/PialEdits_freeview).

MRI scans of poor quality, which showed geometric inaccuracies, were rated “4” (blurred and low quality) or for which the segmentation procedure failed, were excluded.

### Region of interest parcellation

Individual brains were registered on a spherical atlas for parcellation, taking into account individual cortical folding patterns to match brain geometry between the subjects. FreeSurfer parcellated each brain into 148 GM and 32 subcortical ROIs using the Desikan-Killiany-Atlas (56). Afterwards, ROI labels were transformed back into each subject's individual space to compute the volume of each ROI.

### Surface and cortical thickness

Cortical surface area was calculated with FreeSurfer based on a 2D representation of cortical surface after estimation of GM/WM boundary and pial surface (54). Cortical thickness was then calculated for each vertex as distance from the GM/WM boundary and the pial surface. FreeSurfer offers better alignment of cortical landmarks than volume-based registration and does not produce an age-associated bias between older and younger children when registering children's brains to a common space (57).

### Statistical analysis

#### Psychometric data

Group comparisons of demographic and psychometric data (age, IQ, psychometric scores) were carried out using SPSS software, version 22 (IBM Corp., Armonk, NY, USA). We used analysis of variance (ANOVAs) for the assessment of significance of putative differences.

#### Analysis of imaging data

Further analysis of imaging data was carried out using R statistical computing software (58). We tested for differences in cortical GM volumes respecting all regions of FreeSurfer segmentation according to the Desikan-Killiany-Atlas (56). Additionally, we focused on cortical surface parameters in terms of cortical thickness and mean curvature of ROIs, again defined with the Desikan-Killiany-Atlas (56).

We adjusted volume, mean curvature and thickness data for differences in age and IQ using a linear model applying the groups mean age and IQ.

Type III two-way 2 × 2 ANOVAs on the adjusted volume, thickness and curvature data were calculated using the independent between-subject factors ASD diagnosis (yes vs. no) and ADHD diagnosis (yes vs. no; see Table 2). Results were corrected for multiple comparisons applying false discovery rate (FDR) correction. FDR corrected *p* < 0.05 were considered significant, and uncorrected *p* < 0.05 were regarded as trends. We didn't restrict our analysis to a priori regions of interest.

**Table 2:** Factor levels of the 2 × 2 ANOVA model.

	**ADHD diagnosis**	**No ADHD diagnosis**
**ASD diagnosis**	ASD with comorbid ADHD	ASD
**No ASD diagnosis**	ADHD	TD

*TD, typically developed; ASD, autism spectrum disorder; ADHD, attention-deficit/hyperactivity disorder*.

Multiple regression models with either SRS total raw score or DISYPS-II FBB-ADHD as independent variables were conducted with the adjusted left Pars orbitalis volume as dependent variables. The regression model included an interaction term with the binary moderator variables ASD and ADHD.

## Results

### Demographic and psychometric data

Table 3 summarizes the demographic and psychometric data of the ASD, ADHD, and TD group. The study included 95 male participants (6–13 years old): 14 with ASD without comorbid ADHD, 15 with ASD and comorbid ADHD, 30 ADHD, and 36 TD controls. Groups did not differ significantly with respect to age and IQ.

**Table 3:** Demographic and psychometric data.

	**ADHD**	**ASD with comorbid ADHD**	**ASD**	**TD**	**ANOVA**
	**Mean ± *SD***	**Mean ± *SD***	**Mean ± *SD***	**Mean ± *SD***	
N	30	15	14	36	
Age	9.89 ± 2.18	10.32 ± 2.21	10.35 ± 2.47	9.86 ± 2.33	*F*(3,91) = 0.274; *p* = 0.844
IQ	93.71 ± 13.32	98.24 ± 13.37	93.71 ± 14.17	97.79 ± 13.05	*F*(3,91) = 0.789; *p* = 0.503
SRS total score	59.23 ± 31.03	92.93 ± 40.60	81.29 ± 22.10	18.83 ± 14.39	*F*(3,91) = 36.76; *p* < 0.001
DISYPS-II FBB-ADHD	1.44 ± 0.65	1.85 ± 0.61	0.80 ± 0.35	0.20 ± 0.15	*F*(3,64) = 25.25; *p* < 0.001

*N, number; SD, standard deviation; ADHD, attention-deficit/hyperactivity disorder; ASD, autism spectrum disorder; TD, typically developed; IQ, intelligence quotient; SRS, Social Responsiveness Scale; DISYPS, Diagnostik System für Psychische Störungen*.

### Volumetric results

**Figure 1:**
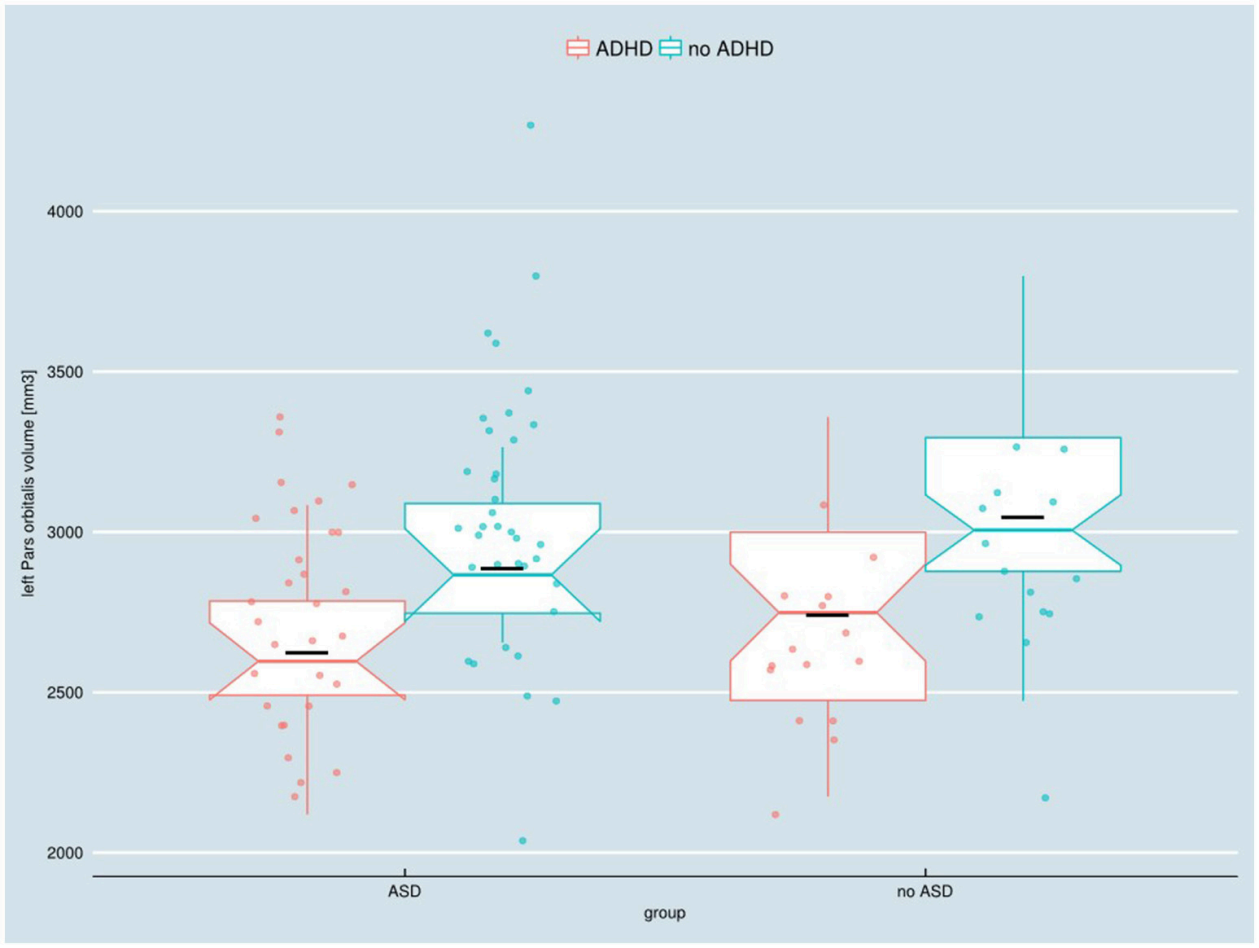
Adjusted left Pars orbitalis volume in children with and without ASD or ADHD, respectively. ADHD, attention-deficit/hyperactivity disorder; ASD, autism spectrum disorder. Boxes indicate upper/lower quartile as well as the median. The black line indicates the sample mean.

The two-way ANOVA model showed a significant main effect of the factor ADHD for the left Pars orbitalis volume after FDR correction [*F*_(1, 91)_ = 12.63; *p*_FDR_ = 0.039, *p*_uncorr_ < 0.001]. Children with an ADHD diagnosis exhibited smaller left Pars orbitalis volumes (Figure 1). Uncorrected significant effects were regarded as trends (Table 4). A main effect of ADHD on trend level could also be observed in the right Pars orbitalis and precuneus cortex, in terms of a volume reduction. An interaction of the factors ADHD and ASD could be detected for the right isthmus cingulate cortex.

**Table 4:** Uncorrected adjusted volume, thickness, and mean curvature results.

**Measure**	**Region**	**ADHD**	**ASD-**	**ASD +**	**TD**	**Two-way ANOVAs**
		**Mean ± *SD***	**Mean ± *SD***	**Mean ± *SD***	**Mean ± *SD***	**(*p*-values)**
Volume	Left pars orbitalis	2738.70 ± 328.53	2884.21 ± 284.83	2621.54 ± 241.09	3043.94 ± 413.61	ASD: *p* = 0.148 **ADHD:** ***p***<**0.001** ASD × ADHD: *p* = 0.784
	Right pars orbitalis	3387.65 ± 485.42	3676.62 ± 613.57	3285.59 ± 463.18	3656.61 ± 430.33	ASD: *p* = 0.896 **ADHD:** ***p*** = **0.027** ASD × ADHD: *p* = 0.572
	Right isthmus cingulate	3204.79 ± 512.29	3244.11 ± 425.46	2875.96 ± 477.66	3075.76 ± 575.98	ASD: *p* = 0.308 ADHD:*p* = 0.320 **ASD** × **ADHD:** ***p*** = **0.035**
	Right precuneus	12868.08 ± 1570.15	13284.51 ± 1850.61	12637.10 ± 1968.18	13783.22 ± 1989.24	ASD: *p* = 0.392 **ADHD:** ***p*** = **0.047** ASD × ADHD: *p* = 0.745
Thickness	Left inferior parietal	2.83 ± 0.14	2.85 ± 0.12	2.70 ± 0.21	2.84 ± 0.14	ASD: *p* = 0.829 ADHD: *p* = 0.708 **ASD** × **ADHD:** ***p*** = **0.039**
	Left post-central	2.24 ± 0.14	2.17 ± 0.12	2.21 ± 0.13	2.31 ± 0.17	**ASD:** ***p*** = **0.004** ADHD: *p* = 0.059 ASD × ADHD: *p* = 0.107
	Right post-central	2.20 ± 0.12	2.11 ± 0.10	2.20 ± 0.14	2.24 ± 0.15	**ASD:** ***p*** = **0.004** ADHD: *p* = 0.218 **ASD** × **ADHD:** ***p*** = **0.030**
	Right superior parietal	2.44 ± 0.14	2.36 ± 0.12	2.37 ± 0.12	2.48 ± 0.14	**ASD:** ***p*** = **0.008** ADHD: p = 0.280 ASD × ADHD: *p* = 0.485
	Left cuneus	2.01 ± 0.18	1.92 ± 0.13	2.00 ± 0.15	2.04 ± 0.16	**ASD:** ***p*** = **0.017** ADHD: *p* = 0.346 ASD × ADHD: *p* = 0.104
	Left parahippocampal	2.89 ± 0.33	2.97 ± 0.29	2.76 ± 0.27	2.84 ± 0.24	ASD: *p* = 0.130 ADHD: *p* = 0.491 **ASD × ADHD:** ***p*** = **0.040**
	Left pars orbitalis	2.89 ± 0.33	2.97 ± 0.29	2.76 ± 0.27	2.84 ± 0.24	ASD: *p* = 0.062 **ADHD:** ***p*** = **0.049** ASD × ADHD: *p* = 0.377
	Left pericalcarine	1.59 ± 0.11	1.54 ± 0.10	1.66 ± 0.19	1.64 ± 0.15	**ASD:** ***p*** = **0.032** ADHD: *p* = 0.177 **ASD × ADHD:** ***p*** = **0.010**
	Left transverse temporal	2.45 ± 0.25	2.39 ± 0.24	2.61 ± 0.22	2.52 ± 0.28	ASD: *p* = 0.124 ADHD: *p* = 0.300 **ASD × ADHD:** ***p*** = **0.016**
Mean curvature	Left transverse temporal	0.141 ± 0.010	0.129 ± 0.010	0.140 ± 0.015	0.133± 0.014	ASD: *p* = 0.304 **ADHD:** ***p*** = **0.012** ASD × ADHD: *p* = 0.544
	Left pericalcarine	0.158 ± 0.016	0.160 ± 0.034	0.150 ± 0.013	0.152 ± 0.014	ASD: *p* = 0.168 ADHD: *p* = 0.179 **ASD × ADHD:** ***p*** = **0.048**
	Left post-central	0.143 ± 0.021	0.149 ± 0.021	0.142 ± 0.016	0.137 ± 0.011	**ASD:** ***p*** = **0.036** ADHD: *p* = 0.188 ASD × ADHD: *p* = 0.100
	Right cuneus	0.165 ± 0.008	0.167 ± 0.013	0.166 ± 0.010	0.161 ± 0.010	**ASD:** ***p*** = **0.016** ADHD: *p* = 0.650 **ASD × ADHD:** ***p*** = **0.018**
	Right medial orbitofrontal	0.147 ± 0.010	0.141 ± 0.006	0.150 ± 0.012	0.148 ± 0.010	**ASD:** ***p*** = **0.016** ADHD: *p* = 0.650 **ASD × ADHD:** ***p*** = **0.018**
	Right pars triangularis	0.144 ± 0.010	0.138 ± 0.011	0.142 ± 0.013	0.139 ± 0.009	ASD: 0.873 **ADHD:** ***p*** = **0.046** ASD × ADHD: *p* = 0.748

*SD, standard deviation; ADHD, attention-deficit/hyperactivity disorder; ASD, autism spectrum disorder; TD, typically developed. Bold: p < 0.05*.

### Cortical thickness

Two-way ANOVA models exhibited no significant main effects or interaction surviving FDR correction. The following uncorrected significant effects were regarded as trends (Table 4):

A main effect for the diagnosis ASD emerged in the bilateral postcentral gyrus and the left pericalcarine and cuneus cortex, as well as in the right superior parietal cortex, in terms of cortical thinning. ADHD effects were observable in the left Pars orbitalis, again, linked to a cortical thinning. An interaction of diagnosis ADHD and ASD could be observed for the left inferior parietal, parahippocampal, pericalcarine, transverse temporal, and right post-central thickness measures.

### Mean curvature

The two-way ANOVA model showed no significant interaction or main effects for the factors ASD or ADHD on mean curvature after FDR correction. Uncorrected significant results we regard as trends (Table 4). The right medial orbitofrontal cortex showed a significant main effect of the factor ASD, as did the left postcentral gyrus and right cuneus cortex. ADHD, in turn, had an effect on mean curvature of the left transverse temporal cortex and right Pars triangularis. Uncorrected significant interactions could be found in the left pericalcarine and right medial orbitofrontal cortex.

### SRS-total score and DISYPS-II effect on main result

Neither the multiple regression model with the independent variable SRS total score nor DISYPS-II FBB-ADHD revealed any significant effect of these scores or their interaction with the factors ADHD and/or ASD on left Pars orbitalis volume.

## Discussion

To our knowledge, this is the first FreeSurfer study that examines children with ADHD, ASD, and comorbid ASD and ADHD in a single study. Our investigation focused on the detection of possible morphometric differences (cortical volume, thickness and mean curvature).

Due to the heterogeneity of findings in earlier studies, we did not limit our analysis to a priori regions of interest.

### Volumetric results

The diagnosis ADHD has a significant effect on the left Pars orbitalis volume with ADHD-diagnosed children showing smaller left Pars orbitalis volumes. These findings suggest that ADHD rather than ASD is related to left Pars orbitalis volume loss. Whether there are weaker “additive” effects on the Pars orbitalis volume of ASD and ADHD cannot be ruled out with a study of the given sample size.

On trend level, we additionally found an ADHD main effect for the right Pars orbitalis and precuneus cortex and an interaction of diagnosis ADHD and ASD for the right isthmus cingulate cortex.

The so-called default-mode network (DMN) has been described as comprising the precuneus/posterior cingulate cortex, the medial prefrontal cortex and the medial, lateral and inferior parietal cortex. It is a network of brain regions associated with task-irrelevant mental processes and mind wandering (59, 60) In line with our results, Castellanos et al. (61) showed ADHD-related decreases in functional connectivity between the precuneus and other DMN components.

### The pars orbitalis of the inferior frontal gyrus

The Pars orbitalis represents a subdivision of the inferior frontal gyrus which more or less corresponds well to the Brodman Area 47.

Functionally it has been linked to the recognition of facial expressions of basic emotions (62) and to the modulation of positive emotionality (63). It is also assumed that besides the specific association between the right inferior frontal gyrus and the inhibitory control, the left inferior frontal gyrus is also involved in the successful implementation of inhibitory control over motor responses (64). This could be partly responsible for the impulsive behavior that can be observed in ADHD.

Children with ADHD and autism have a lot of similar features and there is high frequency of ADHD symptoms in autism (65). Also, in autistic patients, difficulties with emotion and facial recognition have been described (66–69). In addition, it was assumed that Brodmann Area 47 is involved in semantic/syntactic processing (70, 71). Previous studies pointed toward abnormalities in the pragmatic understanding and the use of language in ASD (72). Brothers (73) proposed that there is a network of neural regions (the amygdala, the orbito-frontal cortex, the superior temporal sulcus and gyrus) comprising the “social brain.” Accordingly, reduced Pars orbitalis volume in ASD with comorbid ADHD might not necessarily be responsible for the aforementioned symptoms in ASD, but it may be a complicating factor.

In a recent publication the Pars orbitalis has been implicated as being part of a critical network for the identification of specific ADHD/ASD subtypes (74).

### Relationship to other publications

The volume reduction in the Pars orbitalis is partly consistent with one previous study which reported a trend toward a lower Pars orbitalis volume in ADHD, but in this investigation, the right Pars orbitalis was affected (75). In the present study, decreased right Pars orbitalis volume could only be observed on an uncorrected level (see Table 4). It is assumed that besides the specific association between the right inferior frontal gyrus and the inhibitory control, the left inferior frontal gyrus is also involved in the successful implementation of inhibitory control over motor responses (64). We could not replicate other volumetric results of the few earlier heterogeneous studies that combined ASD and ADHD patients into a single study (3, 10–12, 76). However, it should be mentioned that previous studies are not directly comparable to our study design. Heterogeneous findings are difficult to interpret due to the often arbitrary distinction between both clinical groups without careful consideration of ASD/ADHD comorbidities (74). Furthermore, different methodology in terms of voxel-based morphometry (VBM) was applied in most previous investigations (3, 10, 11) and none of the earlier investigations included ASD– (ASD without comorbid ADHD), ASD+ (ASD with comorbid ADHD), ADHD, and TD participants (3, 10–12).

Additionally, VBM studies examined adolescent samples. Only the study by Mahajan et al. (12) studied children whose average age corresponded to our study (but a smaller age range than our sample). In fact, age and IQ differences across studies are potential factors leading to heterogeneity of results. Even if covariates are used to correct for age effects, the results cannot be transferred to samples from other age groups or age structures.

### Dimensional correlations

Multiple regression models with the independent variable SRS total score or DISYPS-II FBB-ADHD revealed no significant effect of these scores or their interaction with the factors ADHD and/or ASD on left Pars orbitalis volume. It can therefore be assumed that the reduction of the left Pars orbitalis volume is a categorical effect due to ADHD diagnosis and is not due to the severity of different symptoms or traits represented by questionnaires.

### Surface parameters

No significant interaction or main effects for the factors ASD or ADHD on cortical thickness or mean curvature could be detected after FDR correction. The fact that significant group effects only emerged on an uncorrected level might indicate that the effect sizes of possible differences are too small to be detected with the present group size. We decided to interpret the uncorrected significant differences as possible trends.

In doing so, a main effect for ASD diagnosis emerged for the bilateral post-central, left pericalcarine, left cuneus as well as right superior parietal cortical thickness, and an effect for the factor ADHD resulted for the left Pars orbitalis thickness. An interaction of ASD and ADHD diagnosis was detected for left inferior parietal, parahippocampal, pericalcarine, transverse temporal, and right post-central thickness measures.

Our results of cortical thinning in several areas in ASD children (aged 6–13 years) are concordant with studies reporting accelerated thinning in childhood ASD (28). Greater cortical thinning was associated with more severe symptomatology in ASD (31). Another previous study focusing on ASD children (aged 6–12 years) revealed widespread, but mostly left-hemispheric thinning in frontal, temporal, parietal and occipital brain areas related to the theory-of-mind network (77). It should be noted that cortical development in ASD is most likely subject to three different phases: accelerated expansion in early childhood, accelerated thinning in later childhood and adolescence, and decelerated thinning in early adulthood (28). Therefore, when comparing different studies in childhood and adulthood, the exact stage of development must be taken into account.

The detected cortical thinning in the superior parietal cortex in ASD relates well with previous investigations (28, 33). The superior parietal lobule showed decreased activation during learning in ASD and was suggested to play an important role in motor learning and repetitive behaviors (78). Higher SRS- total scores, indicating autistic traits, were associated with thinner cortex in the left superior parietal lobule (79). The postcentral gyrus is important for the representation of haptic and proprioceptive feedback (12). In line with this observation, previous research revealed differences of ASD children in tactile discrimination in comparison to TD (80, 81).

The definite pathomechanisms resulting in cortical thinning are not yet clarified. Regressive (e.g., synaptic pruning) and progressive (e.g., myelination) events are supposed to result in the appearance of GM density reduction or cortical thinning (82), but further research is required.

### Methodological issues and limitations

The study was conducted with a sample of children with ASD or ADHD being prone to motion artifacts (83). Therefore, many of the MRI brain images were excluded from analysis due to poor image quality, which could have biased the study results. We applied manual inspection and correction as suggested by the recommendations of the developers of FreeSurfer. Nevertheless, we cannot completely rule out any confounding effects induced by head motion. Most previous studies did not quantify the degree of observed motion in groups (1). Therefore, differences in the applied (or not applied) motion correction or exclusion criteria might partly be responsible for the heterogeneity of results across studies. To date, a quantification of head motion as described in diffusion tensor imaging (DTI) studies is not possible in FreeSurfer morphometric studies (84). There is very sparse evidence of utilization of automated quality metrics in FreeSurfer studies (85, 86).

Due to the study's focus on primary forms of ASD the results presented here can not necessarily be generalized to forms of ASD with intellectual impairment or to syndromal-secondary autism (defined as autism with known etiology) (87). Additionally, a larger total sample size would have been desirable to detect more potential subtle differences.

Methylphenidate medication was discontinued at least 24 h prior to scanning procedure. Evidence suggests an impact of long-term neurotropic medication on brain structure with stimulant medication being associated with normalization of structural abnormalities in ADHD (88). This confounding factor might have influenced our results, yet, given the earlier literature, not in terms of a volume loss as we report in this study (89). Furthermore, for future studies, it would be helpful to choose longitudinal designs to study longitudinal neurodevelopmental trajectories of ASD and ADHD vs. TD.

## Conclusion

In summary, we detected that ADHD rather than ASD mediates volume loss in the inferior frontal gyrus (Pars orbitalis). The volume reduction in the left Pars orbitalis seems to be primarily a categorical diagnostic effect than to reflect the severity of various traits or symptoms. ASD and ADHD diagnoses tended to have an effect on cortical thickness or mean curvature, which did not survive correction for multiple comparisons. Further studies of more power in larger samples are necessary to investigate the effect of ADHD and ASD on cortical thickness and mean curvature. Additionally, further research is needed to disentangle the precise causal pathways.

## Author contributions

MB is principal investigator of the study funded by German Federal Ministry of Education and Research (BMBF; grant number 01GW0710). MB, JU, RR, IM, CK, and CPK designed the study and were responsible for acquisition and analysis of data. SM, KN, and JM performed FreeSurfer analysis of the data. KN, SM, JM, MB, DE, AR, and LTvE were crucially involved in the theoretical discussion and preparation of the manuscript. KN and SM wrote the manuscript. All authors read and approved the final version of the manuscript. They agreed to be accountable for all aspects of the work.

### Conflict of interest statement

LTvE advisory boards, lectures, or travel grants within the last 4 years: Eli Lilly: Janssen-Cilag, Novartis, Shire, UCB, GSK, Servier, Janssen, and Cyberonics. The remaining authors declare that the research was conducted in the absence of any commercial or financial relationships that could be construed as a potential conflict of interest.
